# Axial heteroatom (P, S and Cl)-decorated Fe single-atom catalyst for the oxygen reduction reaction: a DFT study†

**DOI:** 10.1039/d4ra01754d

**Published:** 2024-05-21

**Authors:** Qian Xue, Xuede Qi, Kun Li, Yi Zeng, Feng Xu, Kai Zhang, Xueqiang Qi, Li Li, Andreu Cabot

**Affiliations:** a College of Chemistry and Chemical Engineering, Chongqing University of Technology Chongqing 400054 China xqqi@cqut.edu.cn; b Catalonia Institute for Energy Research (IREC) Sant Adrià de Besòs Barcelona 08930 Spain acabot@irec.cat; c School of Chemistry and Chemical Engineering, Chongqing University Chongqing 400044 China liliracial@cqu.edu.cn; d ICREA Pg. Lluis Companys 23 08010 Barcelona Catalonia Spain

## Abstract

An FeN_4_ single-atom catalyst (SAC) embedded in a graphene matrix is considered an oxygen reduction reaction (ORR) catalyst for its good activity and durability, and decoration on the Fe active site can further modulate the performance of the FeN_4_ SAC. In this work, the axial heteroatom (L = P, S and Cl)-decorated FeN_4_ SAC (FeN_4_L) and pure FeN_4_ were comparatively studied using density functional theory (DFT) calculations. It was found that the rate-determining step (RDS) in the ORR on pure FeN_4_ is the reduction of OH to H_2_O in the last step with an overpotential of 0.58 V. However, the RDS of the ORR for the axial heteroatom-decorated FeN_4_L is the reduction of O_2_ to OOH in the first step. The axial P and S heteroatom-decorated FeN_4_P and FeN_4_S exhibit lower activity than pure FeN_4_ since the overpotentials of the ORR on FeN_4_P and FeN_4_S are 1.02 V and 1.09 V, respectively. Meanwhile, FeN_4_Cl exhibits the best activity towards the ORR since it possesses the lowest overpotential (0.51 V). The main reason is that the axial heteroatom decoration alleviates the adsorption of all the species in the whole ORR, thus modulating the free energy in every elementary reaction step. A volcano relationship between the d band center and the ORR activity can be determined among the axial heteroatom-decorated FeN_4_L SACs. The d band center of the Fe atom in various FeN_4_L SACs follows the order of FeN_4_ > FeN_4_Cl > FeN_4_S > FeN_4_P, whereas the overpotential of the ORR on various catalysts follows the order of FeN_4_Cl > FeN_4_ > FeN_4_S ≈ FeN_4_P. Δ*G*(*OH) is a simple descriptor for the prediction of the ORR activity of various axial heteroatom-decorated FeN_4_L, although the RDS in the ORR is either the first step or the last step. This paper provides a guide to the design and selection of the ORR over SACs with different axial heteroatom decorations, contributing to the rational design of more powerful ORR electrocatalysts and achieving advances in electrochemical conversion and storage devices.

## Abstract 2

Overpotential and Δ*G*(*OH) have a volcanic relationship, the catalytic process is hindered by too strong or too weak interaction between catalyst and adsorbate. ORR is constrained in strong binding region by the reduction of *OH to H_2_O.

## Graphical abstract

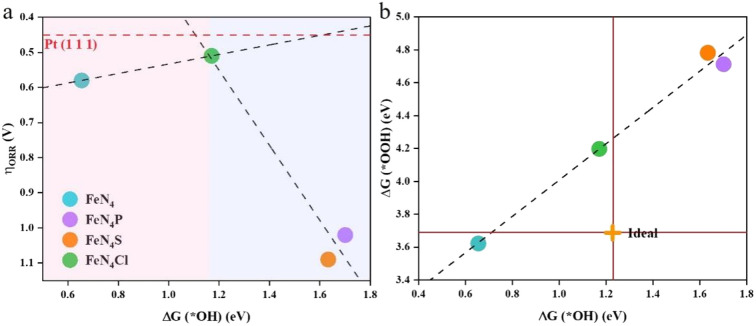

## Introduction

A typical example of a clean energy conversion technology is the proton exchange membrane fuel cell (PEMFC). To ensure energy conversion, the sluggish oxygen reduction reaction (ORR) at the cathode needs a powerful electrocatalyst.^1^ Currently, the most efficient catalyst for the ORR is platinum group metals (PGMs). However, their major drawbacks (such as huge overpotential, scarcity, and high cost) limit their commercialization. One effective way to address this issue is to explore alternatives to non-precious metal catalysts. Carbon materials doped with metals and heteroatoms have drawn much interest from researchers as promising and exceptional electrocatalysts to efficiently reduce the overpotential of the ORR. Overall, heteroatom dopants (such as N, B, P, and S) can produce charged active sites and cause the polarization of the carbon skeleton, which can effectively reduce the overpotential of the ORR and improve the slow kinetics of the ORR.^2–4^ As opposed to PGMs and carbon-based metal-free catalysts, single-atom catalysts (SACs) have emerged as a frontier in the field of ORR electrocatalysts owing to their large specific surface area, maximum atomic efficiency, high selectivity, and long-term operational endurance. DFT calculations have been widely and significantly successful in research studies on the ORR mechanism^5^ since it can greatly save experimental costs and accurately predict the components with high activity. Graphene with defects is one of the numerous substrates that can make an excellent SAC substrate. The electrocatalytic activity of the ORR can be increased by monitoring the d-orbital electrons of the central transition metal (TM) through the coordination of four N atoms,^6^ and the atomically dispersed TMN_4_ site has been considered as the active sites for ORR.^7–9^ Both experimental studies and DFT calculations have shown that the SACs based on non-precious TM (TM = Fe, Co, Mn, Ni, Zn, *etc.*^2,10–12^) on nitrogen-doped carbon support can significantly enhance the ORR electrocatalytic activity. Among the aforementioned SACs, the single-atom Fe–N–C catalyst exhibits considerable activity and remarkable selectivity for the ORR,^9,13,14^ which is even comparable to the commercial platinum catalysts. Furthermore, our previous experiments have proved that the activity and stability of FeN_4_ is as good as the platinum catalyst for ORR.^15,16^ The FeN_4_ catalyst has been reported as a possible replacement for Pt-based catalysts because of its good activity and durability, and the decoration on the Fe active site can further improve the performance of SACs through modulating the electronic and geometric structure.^17,18^ Thus, the rational design of carbon-based ORR catalysts with low cost, as well as high activity, selectivity, and durability, is of great significance. Lin *et al.*^19^ synthesized a new SAC, and each Fe atom is coordinated with five N atoms rather than four N, resulting in a 3D Fe–N_5_ coordination site. By using DFT calculations, they discovered that the axially coordinated pyridine could positively regulate the binding strength of oxygen on the 2D Fe–N_4_ site to a higher level. Other scholars came to similar results that were well-researched.^20,21^ In addition, SACs exhibited good activity for electrochemical N_2_ reduction,^22^ CO_2_ reduction,^23–25^ lithium–sulfur batteries,^26^ NO electroreduction^27,28^ and CO detection.^29^ Thus, the study on axial heteroatom-decorated Fe SAC for ORR is important, and this paper can shed light on the rational design of SAC catalysts with good performance.

In this work, DFT calculations were performed to investigate the effect of different axial heteroatom decorations on the activity of FeN_4_L towards ORR. The binding energy and cohesion energy, which describe the stability of the heteroatom-decorated FeN_4_L, were first studied. Then, the overpotentials of ORR catalyzed by pure FeN_4_ and various FeN_4_L were evaluated. Furthermore, the relationship between the overpotential and the OH adsorption free energy (Δ*G*(*OH)) were studied, since Δ*G*(*OH) is a good descriptor for ORR activity.^30–32^ An accurate quantitative description of the ORR activity trends was elucidated with the help of the adsorption free energy of the reaction intermediate, d-band center, partial density of states, differential charge density and Bader charge of the heteroatom-decorated Fe atom.

## Computational methods

The spin-polarized DFT calculations were performed by using the Vienna *Ab initio* Simulation Package (VASP).^33,34^ The Kohn–Sham wave functions were expanded in a plane wave basis set with a cutoff energy of 500 eV. The projector-augmented wave (PAW) method and PBE potential for the exchange-correlation function were used.^35^ The 3 × 3 × 1 Monkhorst–Pack *k*-point mesh was used to sample the Brillouin zone.^36^ All atoms were allowed to relax until the forces fell below 0.02 eV Å^−1^. A vacuum region of 15 Å was created to ensure negligible interaction between mirror images.

The ORR occurs *via* the following steps:

1 O_2_(g) + * + H^+^ + e^−^ → *OOH

2
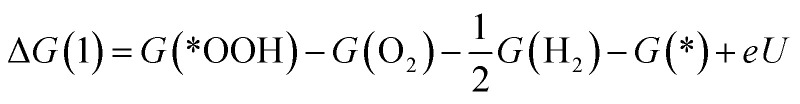

3 *OOH + H^+^ + e^−^ → *O + H_2_O

4
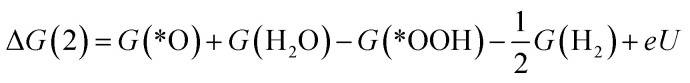

5 *O + H^+^ + e^−^ → *OH

6
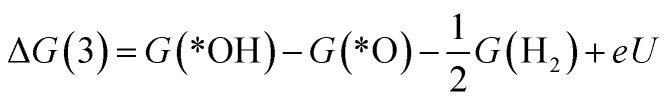

7 *OH + H^+^ + e^−^ → * + H_2_O

8
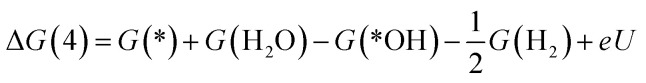
where * represents the preferable adsorption site for intermediates, and *G*(*), *G*(*OOH), *G*(*O) and *G*(*OH) are the Gibbs free energies of the clean catalyst, and the catalyst adsorbed with *OOH, *O and *OH, respectively. *G*(H_2_O) and *G*(H_2_) are the Gibbs free energies of H_2_O and H_2_, respectively.

The Gibbs free energy (Δ*G*) diagram of the ORR was calculated using the computational hydrogen electrode (CHE) model proposed by Nørskov *et al.*,^37^ where the free energy of (H^+^ + e^−^) under standard conditions is equal to the value of 1/2H_2_. The free energy change (Δ*G*) of the primitive step of the ORR is calculated according to the following equation.

9 Δ*G* = Δ*E* + ΔZPE − *T*Δ*S* + Δ∫*C*_P_d*T* + Δ*G*_U_ + Δ*G*_pH_

where Δ*E* is the total energy difference between the reactants and products of the reactions, ΔZPE is the zero-point energy correction, Δ*S* is the vibrational entropy change and *T* is temperature with the value of 298.15 K. Δ∫*C*_P_d*T* is the difference in enthalpic correction, Δ*G*_U_ = −*eU*, where *e* is the elementary charge, *U* is the electrode potential, and Δ*G*_pH_ is the correction of the H^+^ free energy.

The overpotential for ORR (*η*_ORR_) is calculated according to the following equation:

10 *η*_SHE_ = 1.23 V + Δ*G*_max_/*e*

where 1.23 V is defined as the equilibrium potential of the overall 4-electron ORR at the standard state, and Δ*G*_max_ represents the maximum Δ*G* associated with the proton–electron-transfer steps.

The binding energies (*E*_Binding_) of the axial heteroatom with FeN_4_ and the adsorption energy (*E*_ads_) of the intermediates in ORR have been calculated according to formulas (11) and (12):

11 *E*_Binding_ = *E*_FeN_4_X_ − *E*_FeN_4__ − *E*_X_

12 *E*_ads_ = *E*_system_ − *E*_C_ − *E*_species_

where *E*_FeN_4_X_, *E*_FeN_4__ and *E*_X_ are the total electronic energies (non-ZPE corrected) of the system, FeN_4_ and heteroatoms, respectively. *E*_system_, *E*_C_ and *E*_species_ denote the total electronic energy (non-ZPE corrected) of the adsorption system, the individual surface and adsorbate, respectively.

## Result and discussion

A 6 × 6 graphene supercell containing 72 carbon atoms was used as the basis for the construction of the axial heteroatom-decorated FeN_4_ SAC. A double carbon atom vacancy was constructed at the center of graphene. Then, an Fe atom was embedded in the center of the double vacancy, and the four closest C atoms surrounding the vacancy were replaced by four N atoms, resulting in a structural model of the FeN_4_ catalyst.

**Fig. 1:**
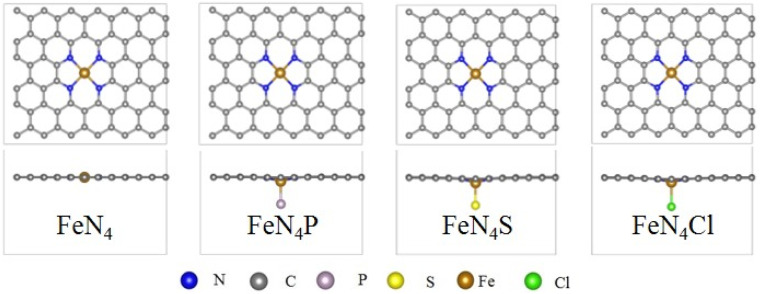
Top and side views of the optimized FeN_4_, FeN_4_P, FeN_4_S, and FeN_4_Cl, respectively.

Fig. 1 shows the top and side views of FeN_4_, FeN_4_P, FeN_4_S, and FeN_4_Cl. The metal and the four coordinated N atoms almost remain in place in the pure FeN_4_, while a slight deviation from the original plane can be determined after the axial heteroatom decoration. The Bader charge in Fig. S1† shows that the central Fe atom in FeN_4_, FeN_4_P, FeN_4_S, and FeN_4_Cl loses electrons (−1.03*e*, −0.86*e*, −1.06*e*, and −1.15*e*; the negative value represents the deficiency of electrons), and electrons are transferred to the surrounding coordinated nonmetal atoms, where the P, S, and Cl atoms get 0.04*e*, 0.44*e*, and 0.54*e*, respectively.

As shown in Table 1, the binding energy between the heteroatom and Fe are all negative with values of −1.79 eV, −3.30 eV, and −2.97 eV, respectively, indicating that these structures can be stabilized. Thus, the subsequent adsorption of the ORR intermediates can be carried out on these structures.

**Table 1:** Binding energy (*E*_Binding_) between the axial heteroatom and Fe in different FeN_4_L

System	FeN_4_P	FeN_4_S	FeN_4_Cl
*E*_Binding_ (eV)	−1.79	−3.03	−2.97

The optimized structures of the *OOH, *O, and *OH intermediates adsorbed on FeN_4_, FeN_4_P, FeN_4_S, and FeN_4_Cl are depicted in Fig. S2.† The Fe atom is located at the center of the double vacant graphene in the FeN_4_, FeN_4_P, FeN_4_S, and FeN_4_Cl systems. The bond lengths between the central Fe and 4 coordinated N atoms are almost similar, and the deviation of the central Fe atom from the graphene plane became smaller compared with the pure FeN_4_. Table 2 lists the bond lengths between the central Fe and various O atoms from the *OOH, *O, *OH intermediates and the inter-bond lengths of various ORR intermediates as well. For the adsorbed *OOH intermediates on various axial heteroatom FeN_4_L, the bond lengths of Fe–O follow the order of FeN_4_S > FeN_4_P > FeN_4_Cl > FeN_4_, and the values are 2.02 Å, 1.95 Å, 1.86 Å, and 1.80 Å, respectively. This is consistent with the fact that the relatively large binding energy usually corresponds to a short distance. For the *O intermediates, a similar phenomenon can also be observed. The bond lengths of Fe–O follow the order of FeN_4_S > FeN_4_P = FeN_4_Cl > FeN_4_, and the values are 1.69 Å, 1.68 Å, 1.68 Å, and 1.65 Å, respectively. Similarly, for the *OH intermediate, the bond lengths of Fe–O follow the order of FeN_4_S > FeN_4_P > FeN_4_Cl > FeN_4_, and the values are 1.94 Å, 1.92 Å, 1.87 Å, and 1.83 Å, respectively. Thus, it can be concluded that the axial heteroatom decoration of FeN_4_L leads to a decreased binding between various ORR intermediates and axial heteroatom-decorated Fe SACs.

**Table 2:** Bond length (Å) between the ORR intermediates OOH, O, and OH and the central active Fe site

System	Bond length/Å					
	*OOH			*O	*OH	
	*d*_Fe–O_	*d*_O–O_	*d*_O–H_	*d*_Fe–O_	*d*_Fe–O_	*d*_O–H_
FeN_4_	1.80	1.47	0.98	1.65	1.83	0.98
FeN_4_P	1.95	1.45	0.98	1.68	1.92	0.98
FeN_4_S	2.02	1.44	0.98	1.69	1.94	0.97
FeN_4_Cl	1.86	1.47	0.98	1.68	1.87	0.98

**Fig. 2:**
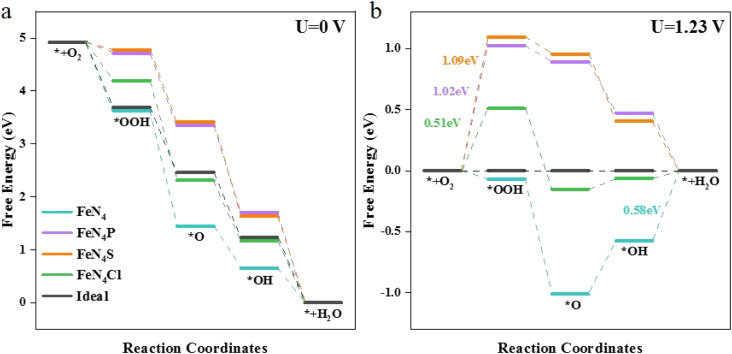
Free energy diagrams for the ORR on FeN_4_, FeN_4_P, FeN_4_S, FeN_4_Cl, and the ideal catalyst at (a) *U* = 0 V and (b) *U* = 1.23 V.

The free energy changes (Δ*G*) on the different catalysts are shown in Fig. 2. The Δ*G* for each elementary step at the active sites of various FeN_4_L are based on the following four reactions: (1) O_2_ adsorbs at the active site and hydrogenates to form *OOH; (2) *OOH continues to hydrogenate to form *O; (3) *O continues to combine with H^+^ and forms *OH; (4) H_2_O is produced through hydrogenation of *OH and eventually desorbs.

At the ORR equilibrium potential, all four Δ*G* values are negative, indicating that each reaction of the ORR is thermodynamically favoured to occur. For a perfect ORR catalyst, Δ*G* should be −1.23 eV for each elementary reaction step involving electron transfer, causing zero overpotential (Fig. 2a). The rate-determining step (RDS) of ORR on FeN_4_ differs from that on the axial heteroatom-decorated FeN_4_L, as illustrated in Fig. 2. The RDS is defined by eqn (10). For FeN_4_, the reaction is determined by the last step of ORR, *i.e.*, the formation of H_2_O from *OH (Δ*G*(4)). However, for the axial heteroatom-decorated FeN_4_P, FeN_4_S, and FeN_4_Cl, the reaction is controlled by the first proton–electron transfer step (Δ*G*(1)), *i.e.*, the protonation of oxygen to *OOH. At 1.23 V, the two steps of hydrogenation of *O and the formation of H_2_O from *OH are free energy increasing processes at the FeN_4_ active site, with the largest free energy increase for *OH. This suggests that the *OH removal step is the RDS with an overpotential of 0.58 V. Whereas the energy of the first step for FeN_4_P, FeN_4_S, and FeN_4_Cl is 1.02 eV, 1.09 eV, and 0.51 eV, respectively. The FeN_4_Cl has the lowest overpotential, indicating that the axial Cl decoration contributed the most to the ORR activity of FeN_4_L compared to the pure FeN_4_ structure, the P and S heteroatom-decorated FeN_4_L.

**Fig. 3:**
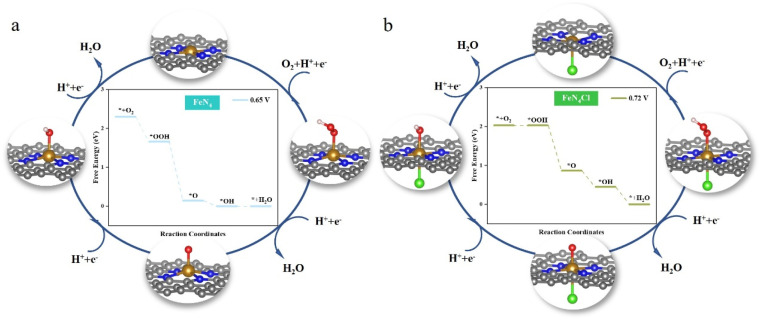
Schematic diagram of the cycle of the ORR. The middle diagram shows Δ*G* of the ORR at different potentials on (a) FeN_4_ and (b) FeN_4_Cl. For *U* < 0.65 V and *U* < 0.72 V, all steps on FeN_4_ and FeN_4_Cl are thermodynamically accessible.

Fig. 3 delves deeper into the ORR catalytic cycle and the optimized conformation of the ORR intermediates on FeN_4_ and FeN_4_Cl to reveal the origin of their high ORR activity (FeN_4_P and FeN_4_S in Fig. S3†). As a transition metal element, the central Fe atom can transfer partial electrons throughout the ORR process, resulting in stronger binding to diverse intermediates. Thus, the obtained conclusion is consistent with the above calculation results, *i.e.*, at the central iron atom, some electron transfer occurs, followed by hydroxide ion desorption. On the contrary, after the central iron atom has been axially decorated with heteroatoms, a portion of the electrons are transferred to the heteroatoms. The electrons provided by the iron atom to the oxygen reduction intermediates are reduced, and the binding becomes weaker, making desorption of the hydroxide ion easier on FeN_4_X. Conversely, the adsorption of O_2_ to generate OOH is the most difficult step in the entire ORR process.

Table 3 demonstrates that the adsorption of various intermediates of the ORR on FeN_4_, FeN_4_P, FeN_4_S, and FeN_4_Cl is an exothermic process. The adsorption of OOH was strongest on the FeN_4_ active site with an adsorption energy of −1.68 eV, moderate adsorption energy of −1.12 eV on FeN_4_Cl, and the smallest adsorption energy on the FeN_4_S surface with a value of −0.53 eV. Meanwhile, it is −0.60 eV on FeN_4_P. Correspondingly, O also exhibited the strongest adsorption on the FeN_4_ active site with −4.36 eV, followed by FeN_4_Cl at −3.52 eV. The adsorption energies on the FeN_4_P and FeN_4_S surfaces are also relatively strong at −2.46 eV and −2.41 eV, respectively. Similarly, the exothermic processes that occur during the OH intermediate adsorption on the surfaces of FeN_4_, FeN_4_P, FeN_4_S, and FeN_4_Cl are all thermodynamically advantageous. *OH has the highest adsorption energy (−2.73 eV) on FeN_4_, the lowest (−1.68 eV) on FeN_4_P, and −1.74 eV and −2.23 eV on FeN_4_S and FeN_4_Cl, respectively. In comparison, OOH, O, and OH on FeN_4_ have the strongest adsorption. Meanwhile, FeN_4_P, FeN_4_S, and FeN_4_Cl, which were decorated by axial heteroatoms, decreased the adsorption of the oxygen-containing ORR intermediates to varying degrees. Furthermore, the adsorption energy of the *O intermediate was higher than both *OOH and *OH on the same catalyst. This indicated that the dissociation of OOH in the first step is relatively simple, and that the hydrogenation reaction of *O occurring in the latter step is also favored by the strong adsorption of the intermediate O.^32^

**Table 3:** The adsorption energy (*E*_ads_) of OOH, O, and OH on FeN_4_, FeN_4_P, FeN_4_S, and FeN_4_Cl

System	Adsorption energy/eV		
	*OOH	*O	*OH
FeN_4_	−1.68	−4.36	−2.73
FeN_4_P	−0.60	−2.46	−1.68
FeN_4_S	−0.53	−2.41	−1.74
FeN_4_Cl	−1.12	−3.52	−2.23

The reaction free energy of the elementary reaction in ORR is determined by the adsorption free energy (Δ*G*_ads_) of the intermediate species, *OH, *O and *OOH (abbreviated as Δ*G*(*OH), Δ*G*(*O), and Δ*G*(*OOH)).^38^ The Δ*G*_ads_ of *OOH, *O, and *OH can be calculated according to the following equations:13
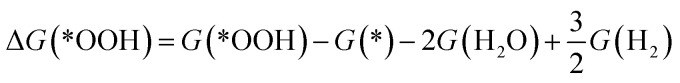

14 Δ*G*(*O) = *G*(*O) − *G*(*) − *G*(H_2_O) + *G*(H_2_)

15
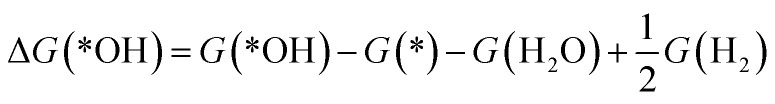


Table S1† lists the calculated Δ*G*(*OH), Δ*G*(*O), and Δ*G*(*OOH) on various catalysts. Δ*G*(*OH) is the variable used to describe the ORR overpotential because RDS is related to either Δ*G*(*OH) or Δ*G*(*OOH). As Δ*G*(*OH) becomes more negative, the strength of the bond between *OH and the catalyst increases.

**Fig. 4:**
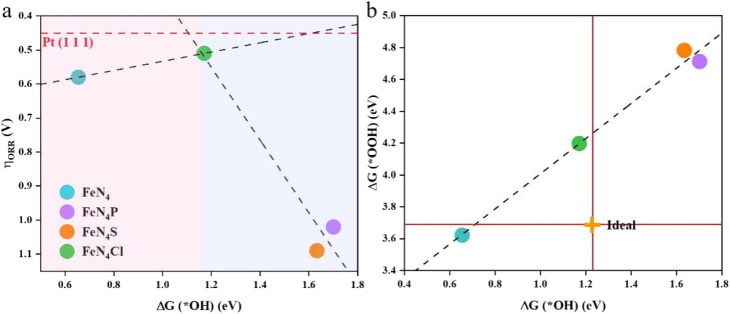
(a) Volcano plot between the theoretical overpotential (*η*_ORR_) and the adsorption free energy of *OH for the ORR on various catalysts. The red dashed line represents the ORR overpotential on Pt(111). (b) The relationship between the adsorption free energy of *OOH and *OH. The ideal intersection point is marked with an orange cross.

The adsorption free energy is not equivalent to the adsorption energy, but reflects the trend of the binding strength of the same substance on different substrates. An appropriate catalyst should have a reasonable Δ*G*_ads_ to make sure that the adsorption of intermediates is neither too high nor too low. The correlation between the ORR overpotential and the Gibbs free energy of the *OH intermediates can be seen in Fig. 4a. The overpotential and Δ*G*(*OH) have a perfect volcanic relationship, and the catalytic process will be hindered by too strong or too weak interactions between the catalysts and intermediates. The inflection point of the volcano diagram is the turning point for both strong and weak adsorption of OH. The ORR process is constrained in the strong binding region (the left pink zone of the volcano diagram) by the reduction of *OH to H_2_O in the last step. The limit of the catalytic process in the weak binding zone (the right blue zone of the volcano diagram) is the first step of O_2_ reduction to *OOH. Among all the studied catalysts here, FeN_4_Cl is nearly at the top of the volcano diagram, and is closest to the theoretical overpotential of Pt(111).^37^ This indicates that FeN_4_Cl possesses a comparable catalytic activity with Pt(111). For the ideal ORR electrocatalyst, Δ*G*(*OH) should be 1.23 eV and Δ*G*(*OOH) should be 3.69 eV. As shown in Fig. 4b, a proportional relationship Δ*G*(*OOH) = 1.10Δ*G*(*OH) + 3.41 with a coefficient of determination *R*^2^ = 0.98 can be determined, indicating a strong linear relationship between the adsorption free energy of *OH and *OOH. The axial heteroatoms decoration of FeN_4_ with Cl, P, and S elements can effectively tune the binding energy of *OH and *OOH, with FeN_4_Cl being closer to the ideal electrocatalyst value than FeN_4_P and FeN_4_S, indicating that the Cl heteroatom decoration can enhance the electrocatalytic activity of FeN_4_ more effectively than both P and S heteroatoms. Thus, the evaluation of ORR activity *via* Δ*G*(*OH) is applicable for the axial heteroatom-decorated FeN_4_L, although the RDS is either the last step for pure FeN_4_ or the first step for various FeN_4_L.

The electronic properties of a substance have a significant impact on its catalytic activity. The study of the electronic properties of catalysts can provide a greater understanding of the origin of the catalytic activity. Therefore, various electronic properties were calculated and analysed to provide insight into the ORR electrocatalytic performances of FeN_4_, FeN_4_P, FeN_4_S, and FeN_4_Cl. The projected density of states (PDOS) was used to evaluate the d-band center of the materials, and the Bader charge and differential charge density of several intermediates were calculated as well.

**Fig. 5:**
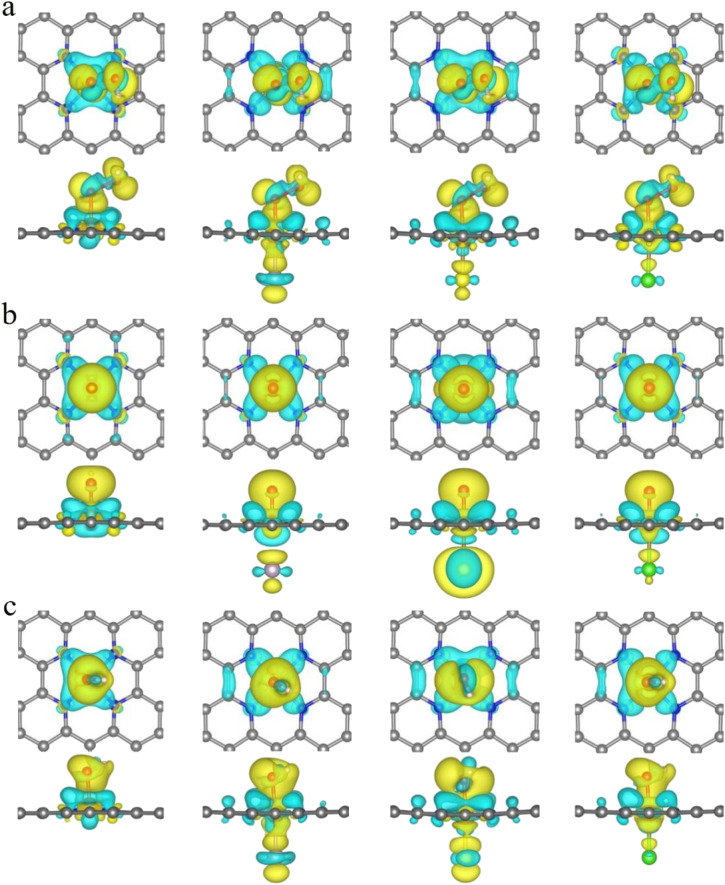
The differential charge density of FeN_4_, FeN_4_P, FeN_4_S and FeN_4_Cl for (a) *OOH, (b) *O and (c) *OH, respectively. The isosurface is 0.002 e Å^−3^.

**Fig. 6:**
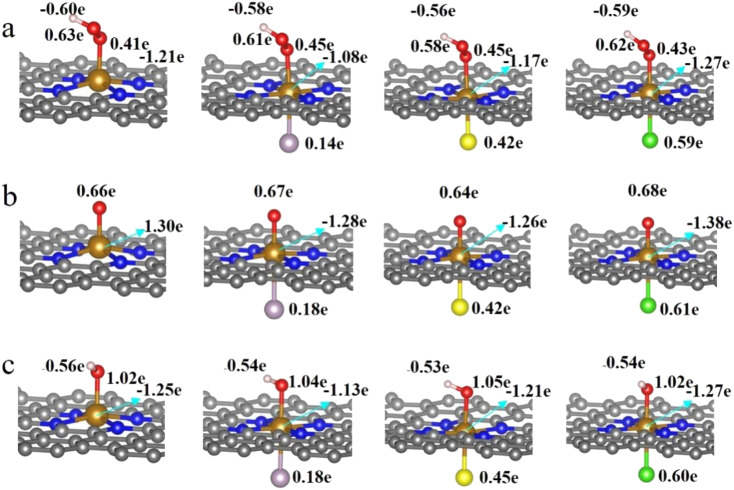
Bader charge analysis of FeN_4_, FeN_4_P, FeN_4_S and FeN_4_Cl for (a) *OOH, (b) *O and (c) *OH, respectively.

It is clear from the results of the differential charge density (Fig. 5) that charge transfer and redistribution occur between the ORR intermediates (*OOH, *O, *OH) and the FeN_4_, FeN_4_P, FeN_4_S, and FeN_4_Cl catalysts. The O atom shows the strongest electronegativity compared to the H, C, N and Fe atoms. Thus, as shown in Fig. 6, in the FeN_4_ system, the O atom receives electrons from the directly attached Fe and/or H atoms, with OH*, O* and OOH* receiving 0.41*e*, 0.66*e* and 1.02*e*, respectively. When FeN_4_ is axially decorated with P, S, and Cl, the electrons obtained by the O atom from the directly attached Fe and/or H atoms are very close to those of FeN_4_, differing only by ±0.01–0.04*e*.

**Fig. 7:**
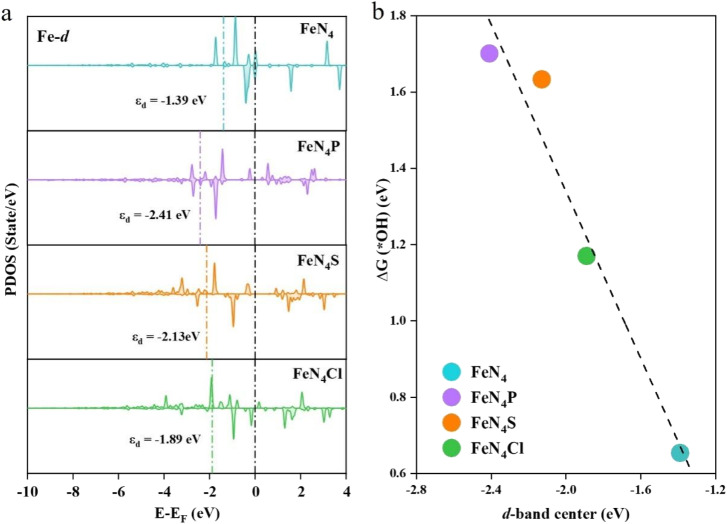
(a) Projected density of states of the Fe atom on FeN_4_, FeN_4_P, FeN_4_S and FeN_4_Cl, and their corresponding d-band centers. (b) Relationship between the d-band center of the Fe atom in FeN_4_, FeN_4_P, FeN_4_S and FeN_4_Cl and Δ*G*(*OH).

The d-bands of the Fe atom in FeN_4_, FeN_4_P, FeN_4_S, and FeN_4_Cl are depicted in Fig. 7a. There are some electrons near the Fermi level; thus, it can be deemed that all of the FeN_4_L SACs possess good conductivity. Furthermore, the d-band center of Fe in FeN_4_ is −1.39 eV, which is closest to the Fermi energy level (*E*_F_) compared with the axial heteroatom-decorated FeN_4_L. The d-band centers of Fe in FeN_4_P, FeN_4_S, and FeN_4_Cl are all farther away from *E*_F_ compared to that in FeN_4_, with the values of −2.41 eV, −2.13 eV and −1.89 eV, respectively. The d-band center theory has successfully described the chemisorption capacity and electrocatalytic properties of transition metals.^39,40^ Generally, a more negative d-band center indicates a weaker adsorption. The d-band center of FeN_4_Cl is closest to *E*_F_ among all the axial heteroatom-decorated FeN_4_L, indicating that FeN_4_Cl is the best catalyst for the ORR intermediate adsorption. Fig. 7b shows the scaling relationship between the d-band center of Fe and Δ*G*(*OH). A negative correlation between the d-band center and Δ*G*(*OH) can be observed, indicating that the intensity of adsorption of OH on Fe atoms increases with the upward shift of the d-band center of the Fe atom, which is consistent with the classical d-band theory.^39,40^

## Conclusions

We performed a detailed calculation and analysis of the structural stability, electronic property, and catalytic activity of both FeN_4_ and axial heteroatom-decorated FeN_4_L (L = P, S and Cl) using DFT calculations. Our results show that all of the axial heteroatom-decorated FeN_4_L SACs have good structural stability. The strong adsorption of the reaction intermediates on FeN_4_ resulted in suboptimal catalytic activity for ORR, and the RDS of ORR on FeN_4_ is the reduction of OH to H_2_O with an overpotential of 0.58 eV. For the axial heteroatom-decorated FeN_4_L, all of the heteroatoms can bind tightly with the central Fe atom in FeN_4_. Meanwhile, the RDS for FeN_4_P, FeN_4_S and FeN_4_Cl is the hydrogenation of O_2_ to OOH with the overpotentials of 1.02 V, 1.09 V and 0.51 V, respectively. FeN_4_Cl is nearly at the top of the volcano diagram, and the overpotential of ORR is closest to Pt(111). The axial Cl heteroatom decoration improves the ORR catalytic activity compared with pure FeN_4_, while the axial P and S heteroatoms have a negative effect on the ORR. The evaluation of ORR activity *via* Δ*G*(*OH) is applicable for the axial heteroatom-decorated FeN_4_L, although the RDS in the ORR is either the first step or the last step.

## Conflicts of interest

There are no conflicts to declare.

## Supplementary Material

RA-014-D4RA01754D-s001
