# Supplementary material for: Axial heteroatom (P, S and Cl)-decorated Fe single-atom catalyst for the oxygen reduction reaction: a DFT study
Source: RSC Adv. 2024 May 21;14(23):16379–88. doi: 10.1039/d4ra01754d (PMC11106811; doi:10.1039/d4ra01754d)
Supplement: RA-014-D4RA01754D-s001 [file RA-014-D4RA01754D-s001.pdf]

Electronic Supplementary Material (ESI) for RSC Advances.
This journal is © The Royal Society of Chemistry 2024

DFT Study of Oxygen Reduction Reaction Catalyzed by Single Fe Atom Catalyst Decorated with Axial Ligand

Qian Xue[a,b], Xuede Qi[a,b], Kun Li[a], Yi Zeng[a], Feng Xu[a], Kai Zhang[a], Xueqiang Qi [a,b,] *, Li Li[c,] *, Andreu Cabot[b,d,*]

[a] College of Chemistry and Chemical Engineering, Chongqing University of Technology, Chongqing 400054, China. Email: xqqi@cqut.edu.cn

[b] Catalonia Institute for Energy Research (IREC), Sant Adrià de Besòs, Barcelona 08930, Spain. Email: acabot@irec.cat

[c] School of Chemistry and Chemical Engineering, Chongqing University, Chongqing 400044, China. Email: liliracial@cqu.edu.cn

[d] ICREA, Pg. Lluis Companys 23, 08010 Barcelona, Catalonia, Spain

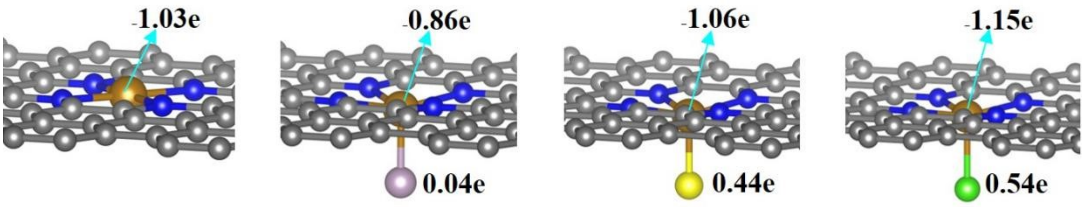

Figure S1 The Bader charge analysis of FeN_4, FeN_4P, FeN_4S, and FeN_4Cl, respectively.

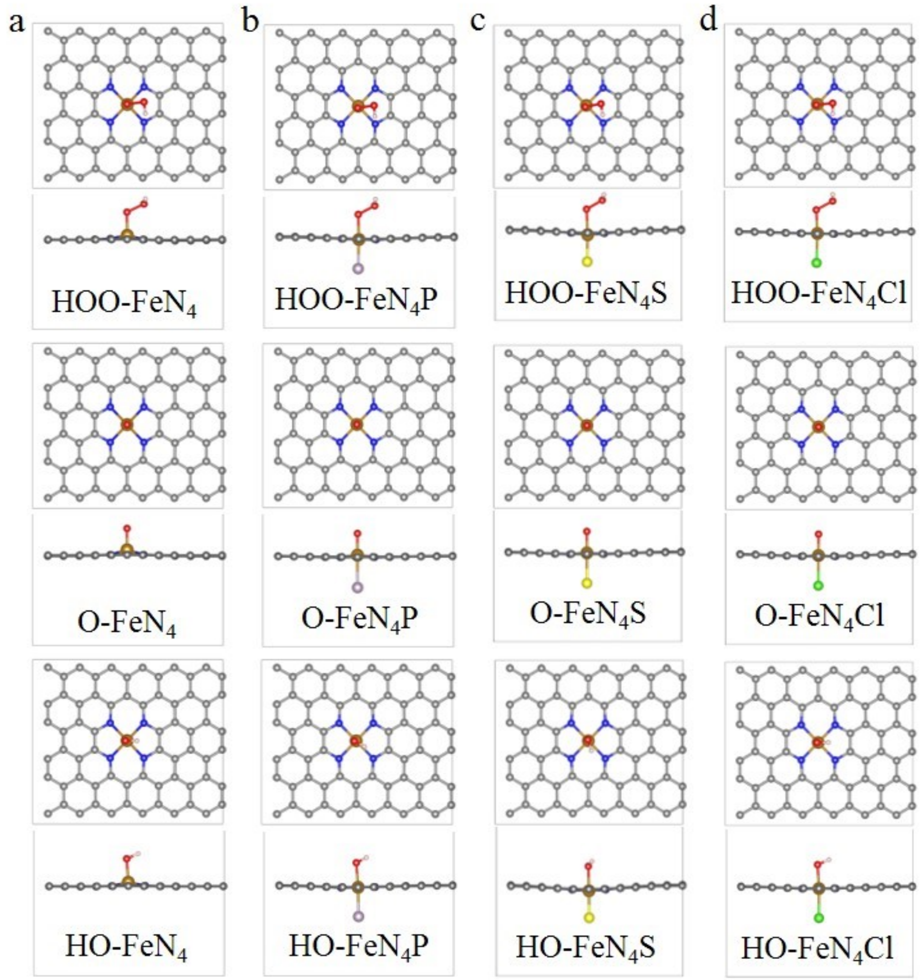

Figure S2 The geometric arrangements of OOH, O, and OH adsorbed on the surfaces of (a) FeN_4, (b) FeN_4P, (c) FeN_4S, and (d) FeN_4Cl during catalytic ORR.

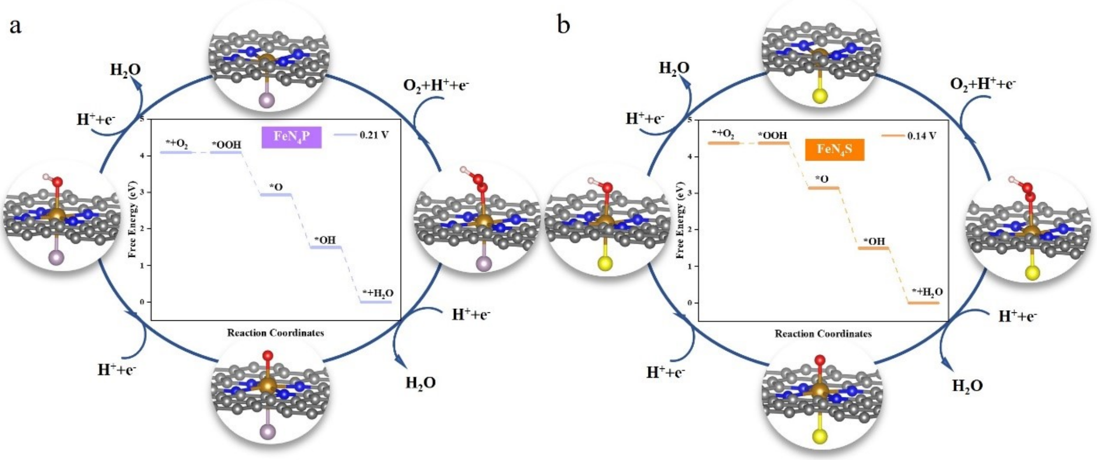

Figure S3 Schematic of the ORR. The inset in the cycle shows the free energy diagram for oxygen reduction reaction on (a) FeN_4P and (b) FeN_4S.

Table S1 The adsorption free energy of ORR intermediates on various catalysts.

System	*Adsorption free energy (ΔG_{ads}) / eV*		
	*OOH	*O	*OH
FeN_4	3.62	1.45	0.65
FeN_4P	4.71	3.35	1.70
FeN_4S	4.78	3.42	1.63
FeN_4Cl	4.20	2.31	1.17
